# Thermal Acclimation to the Highest Natural Ambient Temperature Compromises Physiological Performance in Tadpoles of a Stream-Breeding Savanna Tree Frog

**DOI:** 10.3389/fphys.2021.726440

**Published:** 2021-10-08

**Authors:** Leonardo S. Longhini, Lucas A. Zena, Elias T. Polymeropoulos, Aline C. G. Rocha, Gabriela da Silva Leandro, Cynthia P. A. Prado, Kênia C. Bícego, Luciane H. Gargaglioni

**Affiliations:** ^1^Departamento de Morfologia e Fisiologia Animal, Universidade Estadual Paulista Júlio de Mesquita Filho, Jaboticabal, Brazil; ^2^Department of Physiology, Institute of Biosciences, University of São Paulo, São Paulo, Brazil; ^3^Institute for Marine and Antarctic Studies, University of Tasmania, Hobart, TAS, Australia

**Keywords:** aerobic scope, critical thermal maximum, heart rate, autonomic blockade, climate change, aclimatation, amphibian, oxygen consumption

## Abstract

Amphibians may be more vulnerable to climate-driven habitat modification because of their complex life cycle dependence on land and water. Considering the current rate of global warming, it is critical to identify the vulnerability of a species by assessing its potential to acclimate to warming temperatures. In many species, thermal acclimation provides a reversible physiological adjustment in response to temperature changes, conferring resilience in a changing climate. Here, we investigate the effects of temperature acclimation on the physiological performance of tadpoles of a stream-breeding savanna tree frog (*Bokermannohyla ibitiguara*) in relation to the thermal conditions naturally experienced in their microhabitat (range: 18.8–24.6°C). We quantified performance measures such as routine and maximum metabolic rate at different test (15, 20, 25, 30, and 34°C) and acclimation temperatures (18 and 25°C). We also measured heart rate before and after autonomic blockade with atropine and sotalol at the respective acclimation temperatures. Further, we determined the critical thermal maximum and warming tolerance (critical thermal maximum minus maximum microhabitat temperature), which were not affected by acclimation. Mass-specific routine and mass-specific maximum metabolic rate, as well as heart rate, increased with increasing test temperatures; however, acclimation elevated mass-specific routine metabolic rate while not affecting mass-specific maximum metabolic rate. Heart rate before and after the pharmacological blockade was also unaffected by acclimation. Aerobic scope in animals acclimated to 25°C was substantially reduced, suggesting that physiological performance at the highest temperatures experienced in their natural habitat is compromised. In conclusion, the data suggest that the tadpoles of *B. ibitiguara*, living in a thermally stable environment, have a limited capacity to physiologically adjust to the highest temperatures found in their micro-habitat, making the species more vulnerable to future climate change.

## Introduction

Global warming affects the behavior, distribution, and physiology of many animal species (Parmesan and Yohe, 2003; Parmesan, 2006; Charmantier et al., 2008; Chen et al., 2009; Clusella-Trullas and Chown, 2013; Foden et al., 2013; Settele et al., 2014; Seebacher et al., 2015; Sandblom et al., 2016a; Pacifici et al., 2017). Since the pre-industrial times, the global average temperature has increased by 1.0°C, and during the past decade, record-breaking storms, forest fires, droughts, heat waves, and floods around the world have been documented (IPCC, 2021). It is predicted that extreme weather events and elevated temperature peaks will become more regular in the future (Schär et al., 2004; Diffenbaugh and Ashfaq, 2010) and are likely to influence the performance and survival of a wide range of species globally.

Ectotherms, for instance, are likely to be affected by global warming since many physiological rates such as heart rate and metabolism are strongly influenced by environmental temperature (T_a_). The respiratory and cardiovascular systems are tightly coupled to maintain suitable oxygen delivery to metabolically active tissues, and cardiorespiratory adjustments are generally required whenever metabolic demands change (Overgaard et al., 2012; Hillman and Hedrick, 2015). The effect of T_a_ on metabolic rate typically follows an exponential curve in many ectotherms, roughly doubling for every 10°C increase in T_a_ (i.e., Q_10_ = ∼2, Rocha and Branco, 1998; Overgaard et al., 2012), which is generally accompanied by similar increases in heart rate (*f*_H_) (Bícego-Nahas and Branco, 1999; Hedrick et al., 1999; Seebacher and Franklin, 2011; Overgaard et al., 2012; Zena et al., 2015, 2016). However, many ectotherms remodel their physiology to reduce the extent to which physiological reaction rates change in response to changes in temperature, i.e., thermal acclimation, which is essential for the maintenance of individual performance over a wide range of temperatures (Pough et al., 1992; Rome et al., 1992; Angilletta, 2009; Seebacher et al., 2015). Acclimation may manifest as a reversible change of an organism’s thermal sensitivity when exposed to a new thermal condition, where a physiological rate remains relatively constant despite variations in ambient temperature (Seebacher et al., 2015). For instance, cardiorespiratory functions such as heart rate reset, so that the initially elevated values progressively decrease upon prolonged exposure to moderately high temperatures (Overgaard et al., 2012; Sandblom et al., 2014; Seebacher et al., 2015; Ekström et al., 2016). Such a phenomenon can occur via two mechanisms: (1) reduction of the intrinsic *f*_H_; (2) increase in cholinergic tone and thus reduction of *f*_H_, or even a combination of both. This plasticity of cardiovascular control after prolonged exposure to high T_a_ has already been observed in fish (Ekström et al., 2016; Sandblom et al., 2016b).

Thermal acclimation of metabolic rate and cardiorespiratory functions seem to be crucial for many ectotherms, favoring plastic phenotypes by conferring resilience against predictable (e.g., seasons) and unpredictable changes in T_a_ (Seebacher et al., 2015; Sandblom et al., 2016a). Nevertheless, tropical ectotherms usually experience smaller annual/seasonal changes in environmental temperature, and therefore may be more vulnerable to the impacts of global warming, which bring them closer to their thermal tolerance limits (i.e., difference between minimum [CT_min_] and maximum [CT_max_] critical temperatures) (Somero and DeVries, 1967; Ghalambor et al., 2006; Deutsch et al., 2008; Nilsson et al., 2009; Huey et al., 2012). A lack of comprehensive analyses of the capacity for physiological plasticity across taxonomic groups and geographic regions precludes generalizations regarding thermal plasticity and hence predictions of the impacts of climate change on ectotherms (Simon et al., 2015). According to the International Union for Conservation of Nature (IUCN), more than 50% of amphibian species are susceptible to climate change, and such vulnerability is exacerbated for this particular group of vertebrates since it exhibits several life stages in which normal development requires a contrasting habitat or microhabitat (e.g., water-dependent larval-development with limited dispersal capability) (Foden et al., 2008; Lawler et al., 2010).

Tadpoles are an ideal organism to study thermal physiological adaptations. For instance, their relatively small size and the high heat capacity and thermal conductivity of water make tadpoles virtually isothermal with the environment (Lutterschmidt and Hutchison, 1997). Thus, in consideration of taxonomic as well as geographic diversity, we chose to investigate the thermal acclimation in tadpoles of *Bokermannohyla ibitiguara* (Cardoso, 1983), an endemic anuran amphibian from the Cerrado, a threatened savanna-like morphoclimatic domain in central Brazil (Nali and Prado, 2012). Adults of *B. ibitiguara* are associated with gallery forests, while the tadpoles develop in permanent streams (Haddad et al., 1988; Nali and Prado, 2012; Nali et al., 2020). The significance of the species under consideration is highlighted as “data deficient” by the IUCN (Caramaschi and Eterovick, 2004), and its vulnerability to environmental changes, such as temperature, remains unknown.

We investigated the interacting effects of thermal acclimation (18 vs 25°C) (as a form of phenotypic plasticity) on thermal tolerance and physiological mechanisms of tadpoles of *B. ibitiguara* in relation to recorded T_a_ experienced in the natural habitat. For this purpose, we determined the CT_max_ during acute gradual temperature increases and calculated the warming tolerance (WT, the difference between CT_max_ and maximum temperature found in the micro-habitat). We also evaluated the aerobic scope by measuring routine and maximum metabolic rate at different test temperatures. Additionally, the body characteristics of tadpoles of both acclimation groups were evaluated, and routine *f*_H_ was measured before and after pharmacological autonomic blockade in both groups. Given that some anuran species display mechanisms of thermal compensation (e.g., reset of resting *f*_H_, changes in oxygen consumption or increases of CT_max_), we predicted *B. ibitiguara* tadpoles to display a shift in their thermal tolerance after at least 3 weeks of warm acclimation. Further, warm acclimation and its consequential increase in temperature-induced oxygen demand will result in a chronically altered rate of oxygen consumption and increased capacity for oxygen delivery through modifications in the cardiorespiratory activity, represented by changes in *f*_H_.

## Materials and Methods

### Animal Collection and Maintenance

**FIGURE 1:**
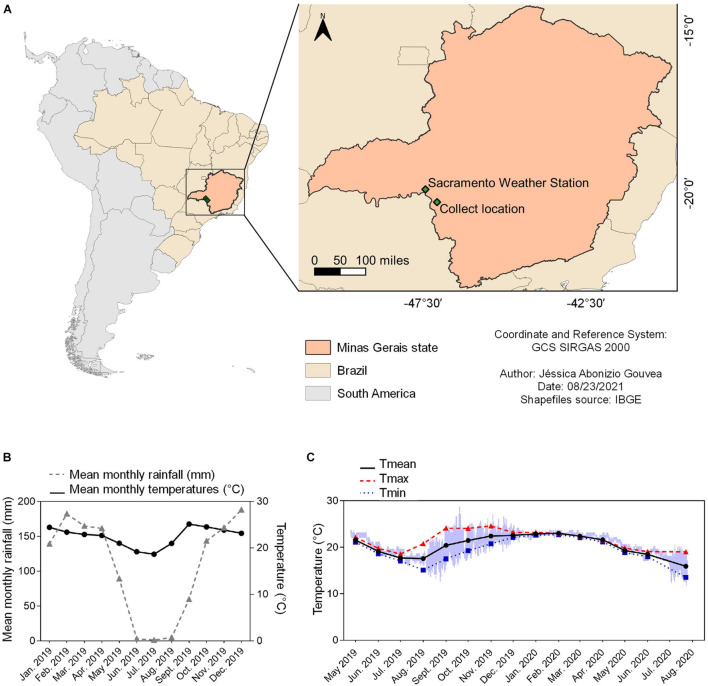
Summary of environmental data for the location that B. ibitiguara inhabits, compared to the weather station. **(A)** Minas Gerais state highlighted on the map of Brazil, showing the capture site of tadpoles of *B. ibitiguara* **(B)** and the nearest weather station **(C)**, at Sacramento city (at a distant of 87.2 km). **(B)** Mean monthly temperatures (black line) and rainfall (blue line) for the year 2019 obtained from the Sacramento weather station. **(C)** Minimum (blue line), maximum (red line) and mean (black line) monthly records of water temperatures in the permanent stream where tadpoles were captured. The data logger collected temperature data every hour from April 24th of 2019 to July 25th of 2020. The time interval estimated for the dried season is from May to September [see graph in panel **(B)**], when, at some point (the total duration is impossible to be determined) the stream displayed decreased water levels and thus and thus, the temperature sensor recorded air temperature.

The anuran species *B. ibitiguara* (Hylidae) is endemic to the Serra da Canastra mountain range in the state of Minas Gerais, southeastern Brazil. Premetamorphic tadpoles (between stages 26 and 30, according to Gosner, 1960; see Table 1 for biometrics) were collected in one semi-permanent stream (Figure 1) located in a rural area, in the municipality of Sacramento (20°16′21.9″S, 47°04′24.5″W; 677 m elevation; Supplementary Figure 1), Minas Gerais state. Using an aquarium fishing net, we collected approximately 25 tadpoles during both day and nighttime on each of the three fieldtrips in February, April and December of 2019. The tadpoles used in the present study originate from different clutches since several adults reproduce in the same stream (Nali and Prado, 2012). Animals were transported in plastic bags to our laboratory at the Department of Animal Morphology and Physiology, UNESP, Jaboticabal, Brazil (approximately 21°14′S and 48°17′W), where they were maintained in two glass aquariums (90 L) under natural photoperiod and temperature set for each acclimation group – 18 and 25°C). Tadpoles did not undergo metamorphoses during any of the experimental protocols. Although the larval period length of *B. ibitiguara* is unknown, stream-breeding species in the genus *Bokermannohyla* are known to exhibit a prolonged larval development phase that may last around 4–5 months (Leite and Eterovick, 2010; Eterovick et al., 2020).

**TABLE 1:** Comparisons of body characteristics of tadpoles in different laboratory acclimation groups (T_acc18_ and T_acc25_°C).

Groups	TL (mm)	PL (mm)	BW (mm)	Body mass (g)
T_acc18_ (*N* = 9)	54.7 ± 1.5	17.9 ± 0.6	9.9 ± 0.5	1.3 ± 0.1
T_acc25_ (*N* = 10)	46.7 ± 1.3	14.8 ± 0.3	7.6 ± 0.3	0.7 ± 0.07
T_acc18_ *vs* T_acc25_	*t*_(17)_ = 3.96; *P <* 0.001	*t*_(17)_ = 4.13; *P <* 0.001	*t*_(17)_ = 3.66; *P <* 0.001	*t*_(17)_ = 4.76; *P <* 0.001

*Values shown are means ± s.e.m. for total body length (TL), partial length (PL), body width (BW) in millimeter (mm) and body mass in grams (g). We tested differences with unpaired t test at a 0.05 significance level. T values and the corresponding degrees of freedom are also shown, all parameters are significantly different between acclimation groups.*

After two days of habituation to the laboratory environment, tadpoles obtained during the first fieldtrip were divided into two acclimation groups, 18 and 28°C. We choose to acclimate tadpoles initially to 28°C in order to test their capacity to tolerate temperatures above the warmest temperature found in their habitat (i.e., 24.6°C); however, all tadpoles exhibited signs of reduced food intake and showed poor body condition. Tadpoles obtained during the second and third fieldtrip were divided into two acclimation groups: 18 and 25°C (hereafter T_acc18_ and T_acc25_, respectively). Therefore, 18°C was chosen because it is coldest temperature that tadpoles may develop in, while 25°C closely represents the warmest condition for *B. ibitiguara* since the maximum temperature found in their habitat was 24.6°C (see section “Microhabitat Temperature”).

For acclimating tadpoles to 18 ± 0.01°C, a stainless-steel coil was positioned inside the aquarium and connected to an external circulation bath via plastic tubes (PolyScience 9112A11B Programmable, Model 9112 Refrigerated Circulator). For acclimating tadpoles to 25 ± 0.02°C, we used a heater controlled by a thermostat (Roxin Ht-1300, 100w) maintained inside the aquarium. Each acclimation temperature was achieved by increasing or decreasing water temperatures by 2°C per day until it reached the desired temperature. All individuals were acclimated at their final treatment temperatures for at least 3 weeks, which is considered a typical acclimation time for small aquatic organisms (Barrionuevo and Fernandes, 1998). Animals were fed daily with herbivore fish food (Maramar, maxi green, 75% vegetable origin). To ensure good water quality, an external filtration system (mechanical, chemical and biological filtration - model HF-0400, Atman, Santo André, São Paulo, Brazil) was used in each aquarium along with an external air pump to maintain water oxygen saturation. Furthermore, twice a week, 20–30% of the aquarium water was removed with animal waste (via a siphon) and replaced with clean water from an artesian well. Thermal gradients inside the aquaria were avoided by creating water motion by the filtration system and the air pumps, and the thermal environment was tested regularly. Animal collection was approved by the Brazilian environmental agency (SISBIO-ICMBio, #621361), and all experimental protocols were approved by the local Animal Care and Use Committee (CEUA-FCAV-UNESP; #02205/18).

### Microhabitat Temperature and Environmental Data

The stream temperature and dissolved oxygen from which tadpoles were collected was recorded for every field trip (four in total: February, April and December of 2019, and July of 2020) at three different sites along the stream. For this, we used a portable dissolved oxygen and temperature polarographic meter (YSI, Model 550A). Additionally, one temperature logger (iButton; Maxim Integrated, San Jose, CA, United States), previously coated in a biologically inert wax mixture (20% Elvax; DuPont, NC, United States; 80% histological paraffin wax), was positioned in the water close to the bottom of the stream, where the tadpoles were found, to record water temperature fluctuations every hour for a year (between April 24th of 2019 and July 25th of 2020). We obtained the mean daily minimum (T_min_), maximum (T_max_) and average (T_mean_) temperatures of the stream water. However, for our final analyses, we considered temperatures recorded only between October and May of 2019, which corresponds to the months of greatest rainfall, consequently with water in the stream, and during the reproductive phase of the species (October–June, Nali and Prado, 2012).

Environmental data were acquired from a weather station located at the Sacramento city, MG, (19°52′48″S, 47°25′48″ W; altitude: 913.12 m) at 87.2 km distance from the stream. The data included daily values for precipitation (mm) and mean ambient temperature (T_mean_; °C) recorded for 2019.

### Body Characteristics

After the acclimation phase, the tadpoles of each group (T_acc18_: *N* = 9; T_acc25_: *N* = 10) were individually weighed on a digital scale (0.01g, Model LW 303i, Bel Engineering, Italy) and measured using calipers (0.01 mm) to obtain the average body mass (BM), total body length (TL, from snout to the end of the tail), partial length (PL, from snout to the insertion of the tail) and body width (BW).

### Upper Thermal Limits

Critical thermal maximum (CT_max_), defined as the thermal point at which activity becomes disoriented, and an animal loses its ability to escape from conditions that lead to death (Cowles and Bogert, 1944), was determined using the dynamic method previously performed in tadpoles (Lutterschmidt and Hutchison, 1997; Duarte et al., 2012; Kern et al., 2015; Agudelo-Cantero and Navas, 2019). The experiment started at the acclimation temperature of each group, then animals were exposed to a constant heating rate of 0.1°C min^–1^ (Supplementary Figure 2) inside a water bath, until we observed immobility after five consecutive taps on the tail using a glass stick (Simon et al., 2015; Badr et al., 2016; Moyano et al., 2017; Agudelo-Cantero and Navas, 2019). The ramp increases in temperature experienced by the tadpoles were continuously measured (sample rate: 1 kHz) using a temperature sensor (MLT415/M Thermistor temperature sensor, ADInstruments^®^, Sydney, Australia). Once an individual reached its CT_max_, we quickly transferred it into a plastic container with water at ∼25°C to allow recovery. Only animals that survived after 24 hours were included in the analysis (T_acc18_: *N* = 7; T_acc25_: *N* = 8 – of the 16 animals tested, only one died within 24 h; the tadpoles for each acclimation groups originated from different collection events).

We also estimated the warming tolerance (WT), which provides a measure of the relative severity of warming that each species can withstand before reaching critical performance levels (Deutsch et al., 2008). This metric was calculated as the difference between the organism’s CT_max_ and the maximum microhabitat temperature (T_max_), i.e., WT = CT_max_ – T_max_) (Duarte et al., 2012). We considered T_max_ to be the mean daily maximum temperature recorded at the stream between October and May of 2019.

### Measuring Oxygen Consumption in Tadpoles

A different sub-sample of tadpoles was used to study the metabolic rates in each acclimation group. The rate of oxygen consumption (≅ metabolic rate = $\dot{M}O_{2}$) was measured in resting tadpoles (T_acc18_: *N* = 8; T_acc25_: *N* = 8) and after forced activity at five test temperatures (15, 20, 25, 30, and 34°C) using fluorescence-based intermittent-flow respirometry (Steffensen, 1989; Clark et al., 2013; Rosewarne et al., 2016; Svendsen et al., 2016). Since it was not possible to keep the tadpoles immobile during respirometry trials, $\dot{M}O_{2}$measurements represent routine metabolic rates (*r*$\dot{M}O_{2}$), indicating the rate of oxygen consumed during low levels of voluntary activity (Fry, 1971; Seebacher and Grigaltchik, 2014).

Each animal was placed in a cylindrical, acrylic respirometer (total volume of 43 mL), submerged in an experimental tank filled with aerated water (PO_2_ = 21 kPa). Through a hole in the upper part of the respirometer, we placed an oxygen sensor (PSt3, PreSens, Regensburg, Germany) and the partial pressure of O_2_ was recorded as per cent of saturation and with a sampling rate of 0.2 Hz using customized software for the O_2_ analyzer (FIBOX3, PreSens, Germany). Inside the experimental tank surrounding the respirometer, an additional aerator was placed to ensure adequate oxygenation of the surrounding water. A submerged recirculation aquarium mini-pump (mini pump A, Sarlobetter, Brazil) was placed within the tank in order to flush the water inside the respirometry chamber. A separate pump (ECEEN, 43GPH), also located within the tank, was used to recirculate water inside the sealed respirometer, and therefore ensure proper mixing for measuring $\dot{M}O_{2}$. Adjustment and maintenance of each test temperature was performed using an external water bath with a coil connected to the experimental tank (PolyScience 9112A11B Programmable, Model 9112 Refrigerated Circulator). The O_2_ sensor was calibrated daily at the test temperatures using 100% aerated distilled water and 0% oxygen by dipping the O_2_ sensor in 100 mL distilled water with 1 g dissolved Na_2_SO_3_ (1% sodium sulphite solution, which acted as an O_2_ scavenger).

Tadpoles were placed into the respirometer for habituation at the first test temperature (15°C) for at least one hour, which is sufficiently long for recovery from handling stress (Kern et al., 2014; Seebacher and Grigaltchik, 2014; Longhini et al., 2017). After one hour, the respirometer was sealed and $\dot{M}O_{2}$ was determined in duplicates at each test temperature (15, 20, 25, 30, and 34°C), always ensuring that O_2_ saturation was kept above 80% (Jensen et al., 2013) during each cycle. At the end of the experimental protocol for measurements of $\dot{M}O_{2}$, tadpoles were removed from the respirometer, and their wet body mass was recorded using digital scales (±0.01 g). Then, animals were transferred to plastic containers with water at ∼25°C. All tadpoles survived the experiments performed for measuring routine metabolic rate.

For measuring maximum metabolic rate (*m*$\dot{M}O_{2}$), we used the manual chasing method immediately before tadpoles were introduced into the respirometer (Clark et al., 2013). This method was chosen because *B. ibitiguara* tadpoles are bottom dwellers, found mostly resting on rocky or silty substrates (Leite and Eterovick, 2010), under or above submerged leaves in the stream. This method makes it possible to achieve *m*$\dot{M}O_{2}$levels due to excess post-exercise oxygen consumption (Reidy et al., 1995; Briceño et al., 2020). For the chasing protocols, a different group of animals (T_acc18_: *N* = 8; T_acc25_: *N* = 8) were placed in a 500 mL beaker inside the same experimental box used for measurements of *r*$\dot{M}O_{2}$. Using a glass stick, we chased the individual for 5 min continuously or until exhaustion occurred (no response after 5 consecutive taps on the tail). After the chasing protocol, tadpoles were immediately placed inside the respirometer that was sealed for measurement of $m\dot{M}O_{2}$. Tadpoles were exposed to the same test temperature (15, 20, 25, 30, and 34°C) and randomly for both acclimation groups). All tadpoles survived the experiments performed for measuring $m\dot{M}O_{2}$, except animals initially tested at 34°C from T_acc25_ (*N* = 2), which represents 11% of total individuals. The respirometry system (acrylic chamber, tubes and pumps) was cleaned daily at the end of each experimental protocol using chlorine to avoid any microbial/algal growth. The background $\dot{M}O_{2}$ was measured in the respirometer without tadpoles as controls, and we subtracted O_2_ consumption of the controls from the experimental values.

The $\dot{M}O_{2}$(μmol g^–1^ h^–1^) during each measurement phase was derived from the slope of the linear regression of O_2_ content (μmol L^–1^) over time (h) according to the equation:


$$\dot{M}O_{2}=V_{RE}W_{o}^{-1}\frac{dCO_{2}}{d\tau }$$

where V_RE_ is the effective volume of water in the respirometer, calculated as the total respirometer volume minus the organism volume, W_o_ is the organism mass (we assumed a density of 1 kg L^–1^) and *dCO*_2_/*dτ* is the slope of the linear decrease in O_2_ content during the time the chamber was sealed (Svendsen et al., 2016). For final $\dot{M}O_{2}$ calculations, we only considered slopes with *r*^2^ ≥ 0.95.

### Drugs

To study the autonomic control of heart rate (*f*_H_), atropine (cholinergic muscarinic antagonist; 3.0 mg kg^−1^) and sotalol (β-adrenergic antagonist; 3.0 mg kg^−1^) were purchased from Sigma-Aldrich (St Louis, MO, United States) and dissolved in amphibian Ringer solution (composition in mmol l^–1^: 46.9 NaCl; 21.0 KCl; 2.40 CaCl; 1.29 MgCl; 3.14 NaHCO_3_; according to Zena et al., 2016; Longhini et al., 2017). Drugs and doses were chosen based on previous studies performed on both tadpole and adult anuran amphibians (Zena et al., 2016; Longhini et al., 2017).

### Heart Rate Measurement and Pharmacological Autonomic Blockade of Heart Rate (*f*_H_)

A different sub-sample of tadpoles was used to study the autonomic control for each acclimation group (T_acc18_: *N* = 8; T_acc25_: *N* = 8). Heart rate was measured using a non-invasive methodology as previously described (Longhini et al., 2017). Briefly, we coupled two parallel electrodes, made from hypodermic needles (40 mm × 1.20 mm, 18G), to a 20 mL plastic syringe positioned inside the experimental tank and connected to a recirculation pump to ensure adequate water exchange between the outside and the inside of the syringe. The electrodes were wired and connected to a signal amplifier (A-M Systems, model 1700, Sequim, WA, United States), allowing the collection of electrical signals from the tadpole’s heart by a direct contact between the electrodes and the animal’s ventral surface. Biological signals were recorded at a sampling rate of 1 kHz by an acquisition system (PowerLab System, ADInstruments^®^, Sydney, Australia) and further analyzed offline (Chart Software, version 7.3, ADinstruments^®^, Sydney, Australia) using the software’s built-in filters (low-pass: 50 Hz) over the raw signals. The online signals were amplified (10.000× gain) and filtered (bandpass: 0.1–5 KHz). The *f*_H_ averages were obtained from 5 minutes of a visibly stable recording that did not contain any obvious artefact resulting from tadpole movements by using the LabChart software’s signal detection tools (version 7.3, Sydney, Australia). In addition, the water system was grounded to attenuate the noise by using a ground wire connected to the amplifier.

The experimental protocol for the blockade of sympathetic and parasympathetic modulation on the heart was initiated after one hour of the tadpoles’ habituation to the experimental apparatus, which was followed by recordings of baseline *f*_H_ measurements for an additional hour. After baseline recordings, tadpoles were gently removed from the experimental apparatus and handled to receive an intraperitoneal injection of atropine. Recording of *f*_H_ occurred for one hour after the muscarinic blockade. Subsequently, sotalol hydrochloride injection was performed to achieve a full autonomic blockade, and *f*_H_ was recorded for an additional hour. Intraperitoneal injections were performed using a dental needle (Mizzy, 200 μm outside diameter) connected by a polyethylene tube (PE-10, Clay Adams, Parsippany, NJ, United States) to a Hamilton syringe (5 μL). Injections were standardized so that the volume injected into the peritoneal cavity was 0.46 μL g^–1^. The autonomic blockade protocol was performed twice in each individual, following an interval of 7 days between the first and the second experiment. At first, the blockage was induced in each individual in their respective acclimation group (T_acc18_ and T_acc25_), that is, at their respective acclimation temperatures, 18 and 25°C. After 7 days, each tadpole was again subjected to the autonomic blockade, but in this case in the form of an acute exposure to the opposite temperature of acclimation, i.e., T_acc18_ was exposed to 25°C for 1 h and T_acc25_ was exposed to 18°C for 1 h before the pharmacological blockade. At the end of the experiments, tadpoles were euthanized by placing them in a solution of benzocaine hydrochloride (250 mg L^–1^) buffered to pH 7.7 with sodium bicarbonate (Longhini et al., 2017). All tadpoles survived to experiments performed for the autonomic blockade, excepted one animal (5%) from T_acc25_, which died during the habituation to the experimental apparatus when acutely exposed to 18°C.

### Statistical Analyses

For comparing the thermal tolerance parameters (CT_max_ and WT) and body characteristics of tadpoles between the two acclimation groups, we used an unpaired *t*-test. To verify the effect of acclimation (T_acc18_ *vs.* T_acc25_), test temperatures (18 *vs.* 25°C), selective autonomic blockade, and their interaction on *f*_H_ (response variable), we fitted linear mixed models by using the R package nlme (Pinheiro et al., 2021). We also fitted linear mixed models for comparing the effects of acclimation (T_acc18_ *vs.* T_acc25_), test temperatures (15, 20, 25, 30, and 34°C) and their interaction on mass-specific *r*$\dot{M}O_{2}$ and *m*$\dot{M}O_{2}$. In all cases, individuals were included as random effects (intercept) to account for the repeatability of the data throughout the study. Absolute aerobic scope (AAS) was calculated as the difference between mean values of *m*$\dot{M}O_{2}$ and *r*$\dot{M}O_{2}$, while the factorial aerobic scope (FAS) was obtained as the ratio of the mean values for *m*$\dot{M}O_{2}$ to *r*$\dot{M}O_{2}$. Factorial and absolute scope were fitted using a Gaussian curve using the Graphpad software, version 8.0.^1^ We also constructed stream temperature frequency histograms of daily values recorded every hour by the data logger, which were bin centered at 0.5 degree interval.

All statistical analyses were performed using R software v. 3.6.3 (R Core Team, 2020). For all analyses, statistical significance was accepted when *P* ≤ 0.05. When significant effects were found in linear models, these were further explored by Tukey’s test for pairwise comparisons within each acclimation treatment. Normality of the residuals were visually inspected by using histograms. Homogeneity of variance for each model was visually inspected and tested using a Levene’s test. When necessary, appropriate data transformations were performed (log transformation).

## Results

### Microhabitat Temperature

Data logger recordings for seasonal temperature changes in the stream where tadpoles of *B. ibitiguara* were collected (sampled between April 24th of 2019 and July 25th of 2020) is shown in Figure 1. During the dry season, we observed that the stream’s flow ceased completely, leaving only non-adjacent pools of stagnant water, which explains the high daily temperature variations between August and November of 2019 (see Figure 1). During our last field trip (July 25th of 2020), we found that the temperature logger was completely emerged from the dried stream bed. By only considering the months in which the stream bed was filled (October–May) according to field observations, T_max_ was 24.6 ± 0.6; T_min_ was 18.8 ± 0.7, while T_mean_ was 21.9 ± 0.8.

For each field trip, we also measured stream water temperature manually at the points where we collected tadpoles, either during daylight or nighttime: February 2019: 24.2°C (15h50); 23°C (16h00) and 22.6°C (8h30); April 2019: 22.5°C (19h30); 22.3°C (20h07) and 22.2°C (10h10); December 2019: 23°C (16h33); 22.7°C (19h48); and July 2020: 21.1°C (12h05); resulting in a T_mean_ of 22.1 ± 0.6. We also measured the dissolved O_2_ in the same location points of collection: 5.6 ± 1.7 mg L^–1^ (range: 4.1–7.3 mg L^–1^; February 2019); 7.7 ± 0.1 mg L^–1^ (range: 7.3–7.8 mg L^–1^; April 2019); 5.7 ± 0.6 mg L^–1^ (range: 4.6–6.7 mg L^–1^; December 2019) and 4.6 ± 0.3 mg L^–1^ (range: 4.1–5.1 mg L^–1^; July 2020).

### Body Characteristics of Acclimation Groups

After the acclimation treatment, all morphological traits were significantly different between T_acc18_ and T_acc25_ (see Table 1). However, none of the acclimation regimes affected the allometric relationships obtained from the residuals of the regressions between total length *vs.* body mass (T_acc18:_ 0.0004 ± 0.3 *vs.* T_acc25_: −0.02 ± 0.3; *t*_(17)_ = 0.05; *P* = 0.96), total length *vs.* partial length (T_acc18:_ 0.009 ± 0.3 *vs.* T_acc25_: 0.008 ± 0.3; *t*_(17)_ = 0.001; *P* = 0.99), and total length *vs.* body width (T_acc18:_ 0.02 ± 0.3 *vs.* T_acc25_: 0.05 ± 0.3; *t*_(17)_ = 0.04; *P* = 0.96).

### Thermal Tolerance

**FIGURE 2:**
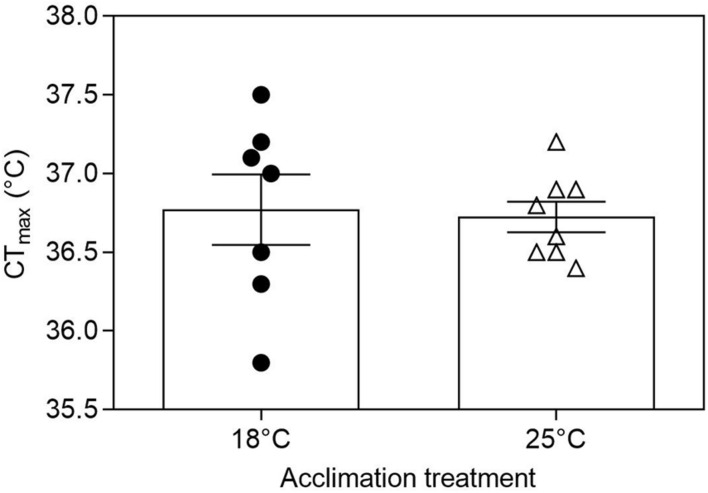
Critical thermal maxima (CT_max_) for tadpoles of *Bokermannohyla ibitiguara*. Acclimation treatment (18 and 25°C) did not affect CT_max_ of premetamorphic tadpoles (*P* = 0.84). Data are presented as mean ± s.e.m. (18°C, *N* = 7; 25°C, *N* = 8).

Tadpoles of *B. ibitiguara* of both acclimation groups exhibited similar CT_max_ (T_acc18_: 36.8 ± 0.2°C *vs*. T_acc25_: 36.7 ± 0.09°C; *t*_(13)_ = 0.19; *P* = 0.84; Figure 2). The heating rate did not differ between the two groups (slope for T_acc18_: 0.089°C min^–1^ *vs.* slope for T_acc25_: 0.091°C min^–1^; F_(1,107)_ = 0.61, *P* = 0.43; see Supplementary Figure 2). WT was also the same for both acclimated groups (T_acc18_: 12.1 ± 0.6°C *vs*. T_acc25_: 12.1 ± 0.2°C; *t*_(1__3__)_ = 0.21; *P* = 0.84).

### Effects of Temperature on Aerobic Metabolism

**FIGURE 3:**
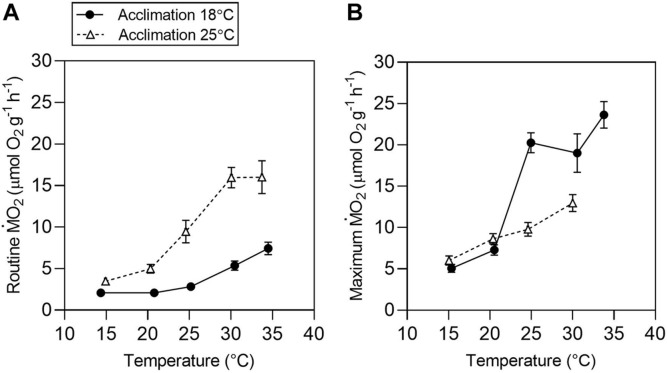
Temperature effects on aerobic metabolism in tadpoles of *Bokermannohyla ibitiguara*. Mass-specific routine [**(A)** *r*$\dot{M}O_{2}]$ and maximum [**(B)** *m*$\dot{M}O_{2}$] metabolic rates for tadpoles acclimated to 18°C (*N* = 8) and 25°C (*N* = 8) and exposed to different test temperatures (15, 20, 25, 30, and 34°C). Data are presented as mean ± s.e.m.

The body mass was significantly different for *r*$\dot{M}O_{2}$ (F_(1,13)_ = 14.43, *P* < 0.001) and *m*$\dot{M}O_{2}$ (F_(1,13)_ = 37.68, *P* = 0.003). In the subsequent analysis, body mass was considered as a possible factor of influence in a covariance analysis. The results for total $\dot{M}O_{2}$ are described in detail in the supplementary material (Supplementary Figure 3). Mass-specific values for *r*$\dot{M}O_{2}$ and *m*$\dot{M}O_{2}$ are shown in Figures 3A,B, respectively. Routine metabolic rate increased with increasing temperature in both acclimation groups (T_acc18_ and T_acc25_) (Test temperature effect: F_(1,62)_ = 302.5, *P* < 0.0001; Figure 3A). Acclimation significantly affected *r*$\dot{M}O_{2}$ (F_(1,14)_ = 68.2, *P* < 0.0001) in tadpoles acclimated to 25°C showing a higher *r*$\dot{M}O_{2}$, with values increasing up to 30°C and showing no further increase when tadpoles were exposed to 34°C (30.06 ± 0.1°C: 15.95 ± 1.2 μmol O_2_ g^–1^ h^–1^ *vs.* 34 ± 0.07°C: 16.01 ± 1.9 μmol O_2_ g^–1^ h^–1^; *t*_(56)_ = 0.221, *P* = 1.0). In contrast, mass-specific *r*$\dot{M}O_{2}$ continues to increase up to 34°C for T_acc18_ (30.4 ± 0.12°C: 5.37 ± 0.5 μmol O_2_ g^–1^ h^–1^ *vs.* 34.4 ± 0.06°C: 7.44 ± 0.7 μmol O_2_ g^–1^ h^–1^; interaction effect: F_(1,62)_ = 4.58, *P* = 0.03). Maximum metabolic rate also increased with increasing test temperature for both T_acc18_ and T_acc25_. The *m*$\dot{M}O_{2}$ of T_acc18_ increased up to 24.9 ± 0.04°C after which no further increase was detected until the temperature reaches 33.7 ± 0.01°C (24.9 ± 0.04°C: 20.2 ± 1.2 μmol O_2_ g^–1^ h^–1^ *vs*. 33.7 ± 0.01°C: 23.6 ± 1.6 μmol O_2_ g^–1^ h^–1^; *t*_(49)_ = −2.267, *P* = 0.43). In contrast, despite a continuous increase in *m*$\dot{M}O_{2}$ for T_acc25_ up to 30 ± 0.05°C, it never reached values similar to T_acc18_ with increasing temperature (interaction effect: F_(1,54)_ = 9.3, *P* < 0.0001; Figure 3B).

**FIGURE 4:**
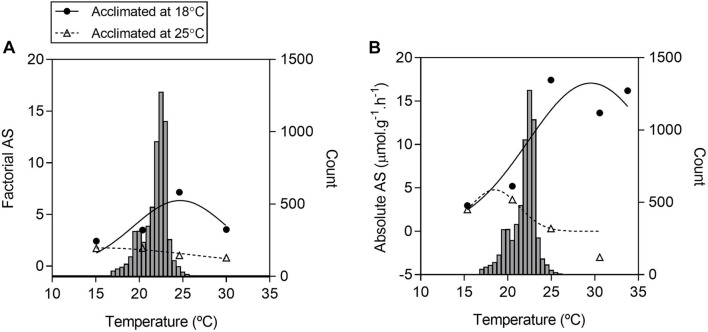
Frequency of water temperature and aerobic scopes of tadpoles of *Bokermannohyla ibitiguara* over a range of test temperatures. Factorial aerobic scope [**(A)** FAS] is calculated from the ratio between mean values obtained for mass-specific maximum metabolic rate (*m*$\dot{M}O_{2}$) and routine metabolic rate (*r*$\dot{M}O_{2}).$ The absolute aerobic scope [**(B)** AAS] is calculated from the difference between mean values for *m*$\dot{M}O_{2}$ and mean values for *r*$\dot{M}O_{2}$ over a range of different temperatures (15 ± 0.06°C to 30 ± 0.05°C). Histograms of the frequency of stream temperature are repeated in panels **(A,B)**, representing the records collected by the data logger every hour between October and May. The right axis indicates the count of records of each water temperature, and the left axis corresponds to the calculated aerobic scope.

Aerobic scope over a range of water temperatures is presented as the absolute difference between mean values of *r*$\dot{M}O_{2}$ and *m*$\dot{M}O_{2}$ (Figure 4B), and as a factorial term calculated as the ratio of the mean values for *m*$\dot{M}O_{2}$ to *r*$\dot{M}O_{2}$ (Figure 4A) with water temperature histograms from the micro-habitat of *B. ibitiguara* measured every hour from the October to May period (with water flow in the stream). For both ways of obtaining the scope, T_acc25_ visually exhibited a smaller amplitude in relation to T_acc18_. In addition to an apparent reduction in aerobic scope for T_acc25_ relative to T_acc18_, the former exhibits maximum values around 20°C, while the latter around 30°C. Furthermore, in T_acc18_ the maximum performance is above the average stream temperature, while the performance is shifted to the left at lower temperatures in T_acc25_.

### Temperature Effects on Heart Rate

**FIGURE 5:**
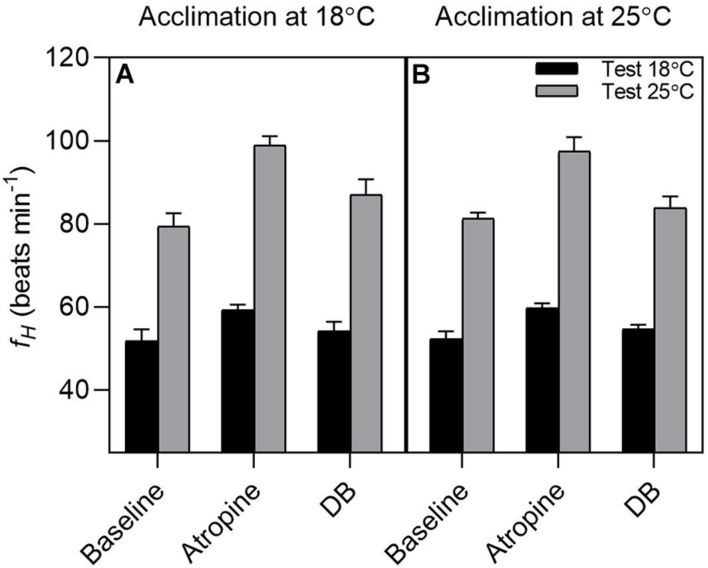
Effect of pharmacological blockade on heart rate in tadpoles of *Bokermannohyla ibitiguara*. The effects of pharmacological blockade (atropine alone and double blockade = atropine + sotalol) on heart rate (*f*_H_) in tadpoles acclimated at 18°C (*N* = 8) and tadpoles acclimated at 25°C (*N* = 7) at different experimental test temperatures (18 and 25°C). Regardless of the treatment treatment used, the *f*_H_ was significantly altered by temperature of 25°C (*P <* 0.001). Data are shown as means ± s.e.m.

Acclimation temperatures did not affect *f*_H_ responses to acute changes in temperature (Acclimation effect: F_(1,12)_ = 0.014; *P* = 0.90; Figure 5), while test temperature significantly affected *f*_H_ (Test temperature effect: F(_1,62)_ = 635.997, *P* < 0.0001). Regardless of the acclimation group, routine heart rate (*f*_H_) increased significantly when acutely exposed from 18 to 25°C (18°C: 52.07 ± 1.6 beats min^–1^ *vs.* 25°C: 80.3 ± 1.6 beats min^–1^; *t*_(62)_ = 12.609, *P* < 0.0001; Figure 5) with a Q_10_ of 1.9.

Pharmacological treatment with atropine and sotalol significantly affected *f*_H_ (treatment effect: F_(2,62)_ = 33.372; *P* < 0.0001). Atropine increased the *f*_H_ of tadpoles at the test temperature of 18°C relative to routine values (atropine: 59.5 ± 0.8 beats min^–1^ *vs.* routine: 52.07 ± 1.6 beats min^–1^; *t*_(62)_ = 3.318, *P* = 0.0182, respectively). Sotalol evoked a slight reduction in *f*_H_, although not significantly different from atropine values (double blockade: 54.4 ± 1.1 beats min^–1^ *vs.* atropine: 59.5 ± 0.8 beats min^–1^; *t*_(62)_ = 2.261, *P* = 0.2257; respectively). When tadpoles were acutely exposed to 25°C, atropine also increased *f*_H_ relative to routine values (atropine: 98.1 ± 1.9 beats min^–1^ *vs.* routine: 80.3 ± 1.6 beats min^–1^; *t*_(62)_ = 7.933, *P* < 0.0001, respectively), although with a larger amplitude effect (interaction effect: F_(2,62)_ = 5.707; *P* = 0.005). Conversely, sotalol evoked a significant reduction in *f*_H_ relative to atropine values (double blockade: 85.5 ± 2.2 beats min^–1^ *vs.* atropine: 98.1 ± 1.9 beats min^–1^; *t*_(62)_ = 5.639, *P* < 0.0001; respectively).

## Discussion

Tadpoles of the anuran *B. ibitiguara* have limited phenotypic plasticity when acclimated to the warmest temperature (∼25°C for at least 3 weeks) found in their micro-habitat. We found that thermal tolerance (i.e., CT_max_) did not differ between acclimation groups (18 and 25°C) and that cardiorespiratory parameters such as routine *f*_H_ and *r*$\dot{M}O_{2}$ increased significantly with high acclimation temperature (i.e., 25°C). Conversely, *m*$\dot{M}O_{2}$ showed a mild increase with acute changes in temperature in tadpoles acclimated to 25°C, thereby remaining low relative to *m*$\dot{M}O_{2}$ values from tadpoles acclimated to 18°C. Therefore, tadpoles exhibited a reduced aerobic metabolic scope when acclimated to 25°C. Our results indicate that *B. ibitiguara* tadpoles are highly susceptible to future events of global warming, in which an average increase of 3°C in the stream temperature that tadpoles inhabit can impact species survival success mainly owing to limited phenotypic plasticity of cardiorespiratory functions.

### Thermal Tolerance of Tadpoles

It is generally expected that species with restricted geographical distributions are exposed to low seasonal temperature variations and therefore show a narrower range of thermal tolerance limits, which may include low capacity for physiological plasticity, such as thermal acclimation (Brattstrom, 1968; Huey and Kingsolver, 1993; Bernardo and Spotila, 2006; Gifford and Kozak, 2012). Chronic acclimation to a high T_a_ of 25°C, did not result in changes to the upper thermal tolerance levels in tadpoles of *B. ibitiguara* from the Cerrado (18°C: 36.8 ± 0.2°C *vs*. 25°C: 36.7 ± 0.09°C). Thus, thermal acclimation appears to be absent. Although a general pattern of increased CT_max_ at relatively high acclimation temperatures has previously been suggested in anurans (Brattstrom, 1968; Navas et al., 2008), some anuran amphibians show a limited scope for or absence of acclimation capacity (Rome et al., 1992; Bovo et al., 2020). Brazilian anuran tadpoles found in contrasting morphoclimatic domains, such as *Rhinella ornata* in the Atlantic forest and *Rhinella granulosa* in drier habitats in the Caatinga exhibit CT_max_ of 42.5 and 44.4°C, respectively (Simon et al., 2015). Tadpoles that develop in ephemeral tropical ponds experience large daily temperature fluctuations and can exhibit CT_max_ above 40°C (Abe and Neto, 1991). In contrast, tadpoles from *B. ibitiguara* exhibit low CT_max_ values, which may result from an adaptation to their micro-habitat that seems to keep low T_a_ oscillations for most part of the year (Figure 1C). *B. ibitiguara* is known to inhabit streams surrounded by gallery forests in a topographically complex landscape at altitudes up to 1.500 m (Nali et al., 2020). In the present study, we sampled tadpoles from a stream at 670 m altitude, a micro-habitat in which there are no large daily or seasonal temperature fluctuations, likely to be related to the presence of gallery forests alongside the streams that the tadpoles inhabit (Supplementary Figure 1).

In order to evaluate the heat-shock risk that tadpoles of *B. ibitiguara* may experience, that is, how fast the tadpole’s performance would decline when approaching the upper thermal limit, we estimated their warming tolerance (Duarte et al., 2012). Since CT_max_ was virtually the same between both acclimation groups, values estimated for warming tolerance were similar and relatively high (T_acc18_: 12.44 ± 0.5°C and T_acc25_:12.42 ± 0.2°C) compared to other tadpole species (Duarte et al., 2012; Simon et al., 2015). This suggests that tadpoles of *B. ibitiguara* tolerate warming before temperatures become deleterious and ultimately lethal, meaning that these tadpoles are in some way resistant to rapid episodes of thermal stress (Duarte et al., 2012; Gutiérrez-Pesquera et al., 2016). Such elevated WT values are in between those recorded for tadpoles living in cool ponds and streams of the subtropical Atlantic Forest in northern Argentina (i.e., WT = 13.2°C; Duarte et al., 2012), and in the Atlantic Forest in southeastern Brazil (i.e., WT = 9.0°C; Simon et al., 2015). In the case of *B. ibitiguara*, adults only reproduce in cool streams that are thermally insulated by gallery forests (Nali and Prado, 2012). Thus the likelihood of long-term thermal heat stress resulting from anthropogenic land-use changes such as deforestation would expose streams to higher daily and seasonal variation in temperature, which may impact the survival of tadpoles. As such, Pintanel et al. (2019) found strong variation in the maximum temperatures in habitats between forests and open environments inhabited by tropical Andean frogs. Their results suggest that environmental thermal variability differences could lead, through local adaptations, to different thermal tolerances. Thus, species tended to be thermal specialists in the less variable thermal environments, similar to what we describe for *B. ibitiguara*.

### Effect of Temperature on Aerobic Metabolism

Previous studies have shown that small aquatic ectotherms may be able to acclimate within a relatively short timeframe (Brown et al., 2004; Rohr et al., 2018). In fact, it is clear that the increase in the stream temperature by 3.1°C relative to the average value (T_mean_: 21.9°C) would considerably impact *B. ibitiguara* tadpoles’ survival success, as tadpoles acclimated at 25°C (T_acc25_) exhibited a relatively high *r*$\dot{M}{\text{O}}_{2}$ compared to tadpoles acclimated at 18°C (T_acc18_). This suggests that *B. ibitiguara* is unable to show thermal compensation of cardiorespiratory functions at 3°C above their habitat’s average temperature (i.e., 21.9°C). In fact, although *r*$\dot{M}{\text{O}}_{2}$ measurements were possible at 34°C in acclimated tadpoles to 25°C, *m*$\dot{M}{\text{O}}_{2}$ measurements at the same test temperature were unsuccessful, as tadpoles did not withstand the chase protocol and some (*N* = 2) died during the initial phase of the subsequent respirometry measurements. Although CT_max_ in T_acc25_ tadpoles was relatively higher (36.7 ± 0.09°C) than the temperature at which tadpoles died (∼34°C), we must consider that tadpoles were warmed relatively fast (i.e., 0.1°C min^–1^). Therefore, we must recognize that the chosen warming protocol to obtain the CT_max_ may have overestimated CT_max_ values, since a slower heating rate could have returned lower CT_max_ values as previously suggested (Chown et al., 2009; Rezende et al., 2011; Ribeiro et al., 2012; Simon et al., 2015).

Noteworthily, in addition to the acclimation temperature at 25°C, we also tested a higher temperature (28°C – tested in tadpoles collected on our first fieldtrip) in which tadpoles were maintained for up to 3 weeks. However, animals exhibited signs of reduced food intake and showed poor body condition, which was also observed in T_acc25_ (visual observation, see Supplementary Figure 4). Other studies have also observed such deleterious effects of high acclimation temperatures in different taxa, such as arthropods, urchins, zooplankton and salmon (Rall et al., 2010; Lemoine and Burkepile, 2012; Alcaraz et al., 2014; Hvas et al., 2017). For instance, Healy and Schulte (2012), studying the fish *Fundulus heteroclitus*, found that at temperatures where both *r*$\dot{M}{\text{O}}_{2}$ and *m*$\dot{M}{\text{O}}_{2}$ were still increasing exponentially with temperature and aerobic scope was maximal, the fish had difficulty maintaining body mass during long-term acclimation. This suggests that there are limitations to the ability to take up, process or assimilate enough nutrients to support the high metabolic rates at high acclimation temperatures (Edwards, 1971; Schulte, 2015).

### Effects of Temperature on Body Size and Developmental Implications

We found significant differences in body measurements between acclimation groups, with T_acc25_ overall, exhibiting smaller body size characteristics compared to T_acc18_ after 3 weeks of acclimation (Table 1). Our data corroborate the decrease in growth observed at the highest acclimation temperature (27°C) in weatherfish larvae of *Misgurnus fossilis* (Schreiber et al., 2017). We recognize our limitations in drawing conclusions about the effect of acclimation temperature on body characteristics due to the lack of data preceding the experiments. However, after the completion of the experimental protocols on aerobic metabolism, tadpoles were returned to their acclimation temperatures, and their further development was observed. Interestingly, the tadpoles from T_acc25_ did not metamorphose, in contrast to individuals of T_acc18_, of which many developed as expected. Normally, environmental stressors such as temperature, prolonged droughts and hypoxic environments would accelerate metamorphosis by increasing the hypothalamus-pituitary-interrenal axis activity (Kikuyama et al., 1993; Owerkowicz et al., 2009; Heinrich et al., 2011; Rollins-Smith, 2017). The putatively reduced growth and the prevention of metamorphosis in tadpoles of T_acc25_ may indicate changes in energy allocation, with most of it being diverted to maintain a high *r*$\dot{M}{\text{O}}_{2}$ (Ruthsatz et al., 2018; Weerathunga and Rajapaksa, 2020). Both thyroid and glucocorticoid hormones are known to trigger metamorphosis in amphibians, and elevated temperatures may activate the hypothalamus-pituitary-interrenal axis and accelerate metamorphosis (Duellman and Trueb, 1994; Crespi and Denver, 2004; Ruthsatz et al., 2018). However, the release of hormones for metamorphosis may demand a high metabolic cost, which could have been disrupted in *B. ibitiguara* tadpoles at 25°C due to the high temperature-driven routine metabolic demand, leading to a trade-off between maintaining body condition or metamorphosis. Interestingly, during a field trip in the middle of the dry season (July 2020), *B. ibitiguara* tadpoles could still be found in what seemed to be permanent water ponds, despite the flow of the stream having ceased. We confirmed that these ponds exhibited a temperature of 21.1°C (time of the day 12h05, similar to the manual measurements obtained in other months) and O_2_ concentration (4.7 mg/L) did not differ from values when the stream had a running flow (see microhabitat values in the results section). Therefore, it seems that *B. ibitiguara* tadpoles can survive through the dry season in suitable thermal conditions by potentially delaying metamorphosis until the following rainy season.

The major weakness in our study stems from the fact that the effects of acclimation on *r*$\dot{M}{\text{O}}_{2}$ and subsequently AAS/FAS cannot be confidently discerned from body size and developmental effects. In particular, the observed increase in *r*$\dot{M}{\text{O}}_{2}$ in T_acc25_ after acclimation could be a result of accelerated development at a higher T_a_, as $\dot{M}{\text{O}}_{2}$ generally increases throughout development (Szdzuy et al., 2008; Sartori et al., 2017). Although we cannot exclude the possibility that the tadpoles did not develop faster (although smaller) than tadpoles in the T_acc18_ group, the results are more supportive of stunted, rather than accelerated growth. Given the lack of information on developmental characteristics in this species and the fact that metamorphosis did not occur in this group, we are confident that the increase in *r*$\dot{M}{\text{O}}_{2}$ is a genuine effect of acclimation, resetting metabolism to an intrinsically higher level and negatively impacting physiological performance and possibly survival. Furthermore, considering global warming will affect most species for many generations, it is important to investigate whether transgenerational and developmental plasticity may allow this species to compensate for climate change, since parental history and egg development may be relevant to the offspring’s thermosensitivity (Seebacher and Grigaltchik, 2014; Donelson et al., 2018).

### Effect of Temperature on Maximum Metabolic Rate

Tadpoles of *B. ibitiguara* are mostly sedentary, unless feeding or escaping from predators, where high levels of $\dot{M}{\text{O}}_{2}$ are required. In regards to the temperature dependency of active oxygen consumption, the *m*$\dot{M}{\text{O}}_{2}$ did not increase much beyond 30°C in T_acc18_ (Figure 3B). Conversely, *r*$\dot{M}{\text{O}}_{2}$ continued its exponential increase in T_acc18_, until the temperature approached a lethal level (34°C), while *r*$\dot{M}{\text{O}}_{2}$ in T_acc25_ reached a plateau at 30°C. This same response was observed in weatherfish larvae (Schreiber et al., 2017) and by Fry (Fry, 1947; Fry and Hart, 1948) when exercising goldfish (*Carassius auratus*), predicting that the optimal temperature for aerobic scope is created by the failure of *m*$\dot{M}{\text{O}}_{2}$ to continue increasing with temperature (Farrell, 2009). Tadpoles of *Limnodynastes peroni* also show an exponential increase in *r*$\dot{M}{\text{O}}_{2}$ (Seebacher and Grigaltchik, 2014). Animals acclimated to the cold (15°C), showed significantly higher O_2_ consumption rates at higher experimental temperatures (20 and 25°C) compared to the group acclimated at higher temperature (i.e., 25°C). In addition, tadpoles of *L. peroni* acclimated to 15°C were more active than animals acclimated to 25°C, which suggests that more oxygen was used by tadpoles acclimated to 15°C for a given level of activity. An alternative explanation is that low temperature activity requires more ATP per unit of muscle power than at high temperature. In our case, both *r*$\dot{M}{\text{O}}_{2}$ and *m*$\dot{M}{\text{O}}_{2}$ increased exponentially in parallel, up to temperatures close to 25°C before the critical maximum temperature that could be tolerated by the tadpoles was reached. Our data corroborates the notion that in more stable environments, such as the stream that the tadpoles of *B. ibitiguara* inhabit, optimal physiological processes may be constrained by a limited range of environmental temperatures (Gabriel, 2005; Gabriel et al., 2005). In addition, as global water temperatures rise, O_2_ solubility in the water is reduced (Dejours, 1981) and therefore animals will face additional challenges to meet the higher oxygen demand of increased metabolic rates (Pörtner et al., 2006).

### Effect of Temperature and Autonomic Blockade on Routine Heart Rate

In this study, the effect of prolonged exposure to elevated temperature, i.e., thermal acclimation, did not cause any compensatory response in the autonomic control of *f*_H_. Thermal acclimation may reset resting *f*_H_ so that the initially elevated *f*_H_ progressively reduces over time upon exposure to the elevated temperature. Such a response is primarily achieved by reducing intrinsic *f*_H_ and/or increasing the inhibitory cholinergic tone on the heart (Haverinen and Vornanen, 2007; Ekström et al., 2016; Sandblom et al., 2016b). The treatment with atropine increased the tadpole’s heart rate at both test temperatures, although the magnitude of the response was temperature dependent, with a more pronounced tachycardia at the higher experimental temperature. In addition, sotalol treatment following atropine reduced *f*_H_ to near baseline values, suggesting routine *f*_H_ and intrinsic *f*_H_ are very similar. This suggests that both cholinergic and adrenergic tone exhibit virtually equal influences on routine *f*_H_. In fact, the lack of acclimation response in the autonomic control of *f*_H_ and intrinsic *f*_H_ in *B. ibitiguara* may be explained by the fact that tadpoles inhabit temperature stable environments. This contrasts with eurythermal species that exhibit thermal acclimation of autonomic control of *f*_H_ with consequent improvements in cardiac function (Seibert, 1979; Sureau et al., 1989; Ekström et al., 2016; Sandblom et al., 2016b).

It is interesting to note that changes in *f*_H_ with acute warming (from 18 to 25°C in T_acc18_) and acute cooling (from 25 to 18°C in the T_acc25_) are equal. However, as previously discussed, routine values for metabolic rate for acclimation group T_acc25_ are considerably elevated relative to acclimation group T_acc18_ (for approximately the same temperature interval, that is, from 20 to 25°C (see Supplementary Figure 5). Since routine *f*_H_ did not differ between acclimation groups, the maintenance of a high routine metabolic rate for T_acc25_ tadpoles can only be explained by increases in cardiac output due to adjustments in stroke volume, and/or increases in arteriovenous extraction. Indeed, increases in stroke volume was previously observed in tadpoles of *Xenopus leavis*, in which significant adjustments in cardiac output after exposure to acute hypoxia occurred by increasing both *f*_H_ and stroke volume (Francis Pan and Burggren, 2013). Yet, this hypothesis remains untested in tadpoles and requires further studies.

## Conclusion and Perspectives

Our study demonstrates that tadpoles of *B. ibitiguara* have a limited phenotypic plasticity in response to acclimation to high temperatures, since the thermal tolerance was not different between acclimation groups (18 and 25°C), and cardiorespiratory functions (i.e., routine *f*_H_ and *r*$\dot{M}{\text{O}}_{2}$*)* increased substantially with high temperature acclimation. On the other hand, *m*$\dot{M}{\text{O}}_{2}$ remained low in relation to the lower temperature of acclimation. Consequently, the tadpoles presented a reduced aerobic metabolic scope when acclimated to a higher temperature (25°C) and therefore an increased vulnerability to climate-driven increases in temperature. In addition, our hypothesis that there would be *f*_H_ compensation due to elevation in cholinergic tone or reductions of intrinsic *f*_H_ was not confirmed, since cholinergic and adrenergic tone exhibit virtually equal influences on resting *f*_H_ independent of acclimation group. These findings may be related to the fact that *B. ibitiguara* tadpoles develop in a stable micro-habitat in which daily and seasonal changes in water temperature are narrow. Such traits may reflect the characteristics of the gallery forests alongside streams that the tadpoles inhabit. Further, tadpoles may find favorable conditions throughout their habitat to allow a prolonged larval phase and possibly adjust the time of metamorphosis to the beginning of the next rainy season.

This lack of plasticity during the larval phase of *B. ibitiguara* has important conservation implications, because adults of this anuran amphibian are habitat specialists, always associated to a topographically complex landscape that has endured anthropogenic modification (Nali et al., 2020). Moreover, in recent years the region where the study was conducted has experienced prolonged droughts and streams have been used to capture water, contributing to more frequent drying periods. Also, the Brazilian Cerrado is one of the most threatened tropical savannas in the world, with nearly a 100 endemic amphibians’ species, including *B. ibitiguara* (Nali and Prado, 2012; Valdujo et al., 2012; CEPF: Critical Ecosystem Partnership Fund, 2017). Therefore, in a scenario with prolonged droughts, gradual increases in ambient temperatures and degradation of remaining gallery forests in non-protected areas of the Brazilian Cerrado, the survival of this species will likely be affected. Thus, even if CT_max_ values found here are above the temperatures the species usually experience, the probability of experiencing high temperatures above their optimal temperatures would increase in the future. In addition, there are very few studies on this topic, despite the enormous diversity of anuran species in Brazil and in the Neotropical region.

## Data Availability Statement

The raw data supporting the conclusions of this article will be made available by the authors, without undue reservation.

## Ethics Statement

The animal study was reviewed and approved by animal collection was approved by the Brazilian Environmental Agency (SISBIO-ICMBio, #621361), and all experimental protocols were approved by the local Animal Care and Use Committee (CEUA-FCAV-UNESP; #02205/18).

## Author Contributions

LSL, LAZ, ETP, and LHG designed the research. LSL, LAZ, ACGR, and GSL performed the experiments and LSL, LAZ, and ETP analyzed the data. LAZ and LHG supervised the project. All authors interpreted the data and provided critical and intellectual input during the preparation of the manuscript and approved the final version.

## Conflict of Interest

The authors declare that the research was conducted in the absence of any commercial or financial relationships that could be construed as a potential conflict of interest.

## Publisher’s Note

All claims expressed in this article are solely those of the authors and do not necessarily represent those of their affiliated organizations, or those of the publisher, the editors and the reviewers. Any product that may be evaluated in this article, or claim that may be made by its manufacturer, is not guaranteed or endorsed by the publisher.

## Footnotes

1
www.graphpad.com
