# Supplementary material for: Thermal Acclimation to the Highest Natural Ambient Temperature Compromises Physiological Performance in Tadpoles of a Stream-Breeding Savanna Tree Frog
Source: Front Physiol. 2021 Oct 8;12:726440. doi: 10.3389/fphys.2021.726440 (PMC8531205; doi:10.3389/fphys.2021.726440)
Supplement: Supplementary file 1 [file Data_Sheet_1.docx]

Supplementary Material

**Material and methods**

## Measuring oxygen consumption in tadpoles

A different sub-sample of tadpoles were used to study the metabolic rates in each acclimation group. The rate of oxygen consumption (≅ metabolic rate = $\dot{M}O_{2}$) was measured in resting tadpoles (T_acc18_: N=8; T_acc25_: N=8) and after forced activity at five test temperatures (15, 20, 25, 30 and 34°C) using fluorescence-based intermittent-flow respirometry (Steffensen, 1989; Clark et al., 2013; Svendsen et al., 2016; Rosewarne et al., 2016). Since it was not possible to keep the tadpoles immobile during respirometry trials, $\dot{M}O_{2}$measurements represent routine metabolic rates (*r*$\dot{M}O_{2}$), indicating the rate of oxygen consumed during low levels of voluntary activity (Fry, 1971; Seebacher and Grigaltchik, 2014).

Each animal was placed in a cylindrical, acrylic respirometer (total volume of 43 mL), submerged in an experimental tank filled with aerated water (PO_2_=21 kPa). Through a hole in the upper part of the respirometer, we placed an oxygen sensor (PSt3, PreSens, Regensburg, Germany) and the partial pressure of O_2_ was recorded as percent of saturation and with a sampling rate of 0.2 Hz using customized software for the O_2_ analyzer (FIBOX3, PreSens, Germany). Inside the experimental tank surrounding the respirometer, an additional aerator was placed to ensure adequate oxygenation of the surrounding water. A submerged recirculation aquarium mini-pump (mini pump A, Sarlobetter, Brazil) was placed within the tank in order to flush the water inside the respirometry chamber. A separate pump (ECEEN, 43GPH), also located within the tank, was used to recirculate water inside the sealed respirometer, and therefore ensure proper mixing for measuring $\dot{M}O_{2}$. Adjustment and maintenance of each test temperature was performed using an external water bath with a coil connected to the experimental tank (PolyScience 9112A11B Programmable, Model 9112 Refrigerated Circulator). The O_2_ sensor was calibrated daily at the test temperatures using 100% aerated distilled water and 0% oxygen by dipping the O_2_ sensor in 100 mL distilled water with 1 g dissolved Na_2_SO_3_ (1% sodium sulphite solution, which acted as an O_2_ scavenger).

Tadpoles were placed into the respirometer for habituation at the first test temperature (15°C) for at least one hour, which is sufficiently long for recovery from handling stress (Kern et al., 2014; Seebacher and Grigaltchik, 2014; Longhini et al., 2017). After one hour, the respirometer was sealed and $\dot{M}O_{2}$ was determined in duplicates at each test temperature (15, 20, 25, 30 and 34°C), always ensuring that O_2_ saturation was kept above 80% (Jensen et al., 2013) during each cycle. At the end of the experimental protocol for measurements of $\dot{M}O_{2}$, tadpoles were removed from the respirometer and their wet body mass recorded using digital scales (±0.01 g). Then, animals were transferred to plastic containers with water at ~25°C. All tadpoles survived to experiments performed for measuring routine metabolic rate.

For measuring maximum metabolic rate (*m*$\dot{M}O_{2}$), we used the manual chasing method immediately before tadpoles were introduced into the respirometer (Clark et al., 2013). This method was chosen because *B. ibitiguara* tadpoles are bottom dwellers, found mostly resting on rocky or silty substrates (Leite and Eterovick, 2010), under or above submerged leaves in the stream. This method makes it possible to achieve *m*$\dot{M}O_{2}$ levels due to excess post-exercise oxygen consumption (Reidy et al., 1995; Briceño et al., 2020). For the chasing protocols, a different group of animals (T_acc18_: N=8; T_acc25_: N=8) were placed in a 500 mL beaker inside the same experimental box used for measurements of *r*$\dot{M}O_{2}$. Using a glass stick, we chased the individual for 5 minutes continuously or until exhaustion occurred (no response after 5 consecutive taps on the tail). After the chasing protocol, tadpoles were immediately placed inside the respirometer that was sealed for measurement of $m\dot{M}O_{2}$. Tadpoles were exposed to the same test temperature (15, 20, 25, 30 and 34°C) and randomly for both acclimation groups). All tadpoles survived to experiments performed for measuring $m\dot{M}O_{2}$, except animals initially tested at 34°C from T_acc25_ (N=2). The respirometry system (acrylic chamber, tubes and pumps) was cleaned daily at the end of each experimental protocol using chlorine to avoid any microbial/algal growth. The background $\dot{M}O_{2}$ was low and never exceeded 5% of the tadpole’s O_2_ consumption.

The $\dot{M}O_{2}$ (μmol g^-1^ h^− 1^) during each measurement phase was derived from the slope of the linear regression of O_2_ content (µmol L^-1^) over time (h) according to the equation:

$$\dot{M}O_{2}=V_{RE}W_{o}^{-1}\frac{d{CO}_{2}}{d\tau}$$

where V_RE_ is the effective volume of water in the respirometer, calculated as the total respirometer volume minus the organism volume, W_o_ is the organism mass (we assumed a density of 1 kg L^−1^) and 𝑑𝐶𝑂_2_/𝑑𝜏 is the slope of the linear decrease in O_2_ content during the time the chamber was sealed (Svendsen et al., 2016). For final $\dot{M}O_{2}$ calculations, we only considered slopes with r^2^ ≥ 0.95.

## Statistical analyses

We fitted linear mixed models by using the R package nlme (Pinheiro et al., 201) to compare the effects of acclimation (T_acc18_ *vs.* T_acc25_), test temperatures (15, 20, 25, 30 and 34°C) and their interaction on total *r*$\dot{M}O_{2}$ and *m*$\dot{M}O_{2}$. These analyses were performed by taking the tadpole’s body mass as a co-variable into consideration. In all cases, the individual was included as a random effect (intercept) to account for the repeatability of the data throughout the study.

All statistical analyses were performed using R software v. 3.6.3 (R Core Team, 2020). For all analyses, statistical significance was accepted when *P* ≤ 0.05. Normality of the residuals were visually inspected by using histograms. Homogeneity of variance for each model was visually inspected and tested using a Levene’s test. When necessary, appropriate data transformations were performed (log transformation).

# Results

**2.1 Effects of temperature on aerobic metabolism**

The total *r*$\dot{M}O_{2}$ and *m*$\dot{M}O_{2}$ are represented in figures S3A and S3B, and the results for routine are the same as those obtained for mass-specific *r*$\dot{M}O_{2}$, with significance for test temperature (F_(1,62)_= 302.5, *P* < 0.0001), acclimation (F_(1,13)_= 80.5, *P* < 0.0001) and the interaction between both (F_(1,62)_= 4.57, *P* = 0.03), as well as the mass (F_(1,13)_= 14.4, *P* < 0.0001; Fig. S3A). In relation to total *m*$\dot{M}O_{2}$, we observed a difference in the lower test temperatures (15 and 20°C) compared to the values for mass-specific *m*$\dot{M}O_{2}$, in which T_acc25_ had higher values than T_acc18_. However, the same factors were significant in both cases (Test temperature effect: F_(1,54)_= 177.5, *P* < 0.0001 and interaction between acclimation and test temperature: F_(1,54)_= 9.2, *P* < 0.0001; Fig. S3B). The body mass was also significantly different for *m*$\dot{M}O_{2}$ (F_(1,13)_= 37.6, *P* < 0.0001), showing the need for correction of the $\dot{M}O_{2}$values by the mass.

# References

Briceño, F. A., Fitzgibbon, Q. P., Polymeropoulos, E. T., Hinojosa, I. A., and Pecl, G. T. (2020). Temperature alters the physiological response of spiny lobsters under predation risk. *Conservation Physiology* 8, 1-16.

Clark, T. D., Sandblom, E., and Jutfelt, F. (2013). Aerobic scope measurements of fishes in an era of climate change: respirometry, relevance and recommendations. *J. Exp. Biol.* 216, 2771-2782.

Fry, F. E. J. (1971) The effect of environmental factors on the physiology of fish. In WS Hoar, DJ Randall, eds, Fish Physiology. Academic Press, San Diego, pp. 1–99.

Jensen, M. A., Fitzgibbon, Q. P., Carter, C. G., and Adams, L. R. (2013). Recovery periods of cultured spiny lobster, *Sagmariasus verreauxi* juveniles: effects of handling, force feeding, exercising to exhaustion and anaesthesia on oxygen consumption and ammonia-N excretion rates. *Aquaculture* 410–41, 114–121.

Kern P, Cramp R. L., and Franklin C. E. (2014). Temperature and UV-B-insensitive performance in tadpoles of the ornate burrowing frog: an ephemeral pond specialist. *J. Exp. Biol.* 217, 1246–1252.

Leite, F. S. F, and Eterovick, P. C. (2010). Description of the tadpole of *Bokermannohyla martinsi* (Anura: Hylidae), morphological and ecological comparison with related *Bokermannohyla* tadpoles. *Journal of Herpetology* 44, 3, 431–440.

Longhini, L. S., Zena, L. A., da Silva, G. S. F., Bícego, K. C., and Gargaglioni, L. H. (2017). Temperature effects on the cardiorespiratory control of American bullfrog tadpoles based on a non-invasive methodology. *J. Exp. Biol.* 220, 3763-3770.

Reidy, S. P., Nelson, J. A., Tang, Y. Y., and Kerr, S. R. (1995). Post-exercise metabolic rate in Atlantic cod and its dependence upon the method of exhaustion. *J. Fish Biol.* 47, 377-386.

Rosewarne, P. J., Wilson, J. M., and Svendsen, J. C. (2016). Measuring maximum and standard metabolic rates using intermittent-flow respirometry: a student laboratory investigation of aerobic metabolic scope and environmental hypoxia in aquatic breathers. *J.Fish Biol.* 88, 265–283.

Seebacher, F., and Grigaltchik, V. S. (2014). Embryonic developmental temperatures modulate thermal acclimation of performance curves in tadpoles of the frog *Limnodynastes peronei*. *PLoS ONE* 9, 1-11.

Steffensen, J. (1989) Some errors in respirometry of aquatic breathers: how to avoid and correct for them. *Fish Physiol Biochem* 6, 49–59.

Svendsen, M. B. S., Bushnell, P. G., and Steffensen, J. F. (2016). Design and setup of intermittent-flow respirometry system for aquatic organisms. *J. Fish Biol.* 88, 26–50.

# Supplementary Figures


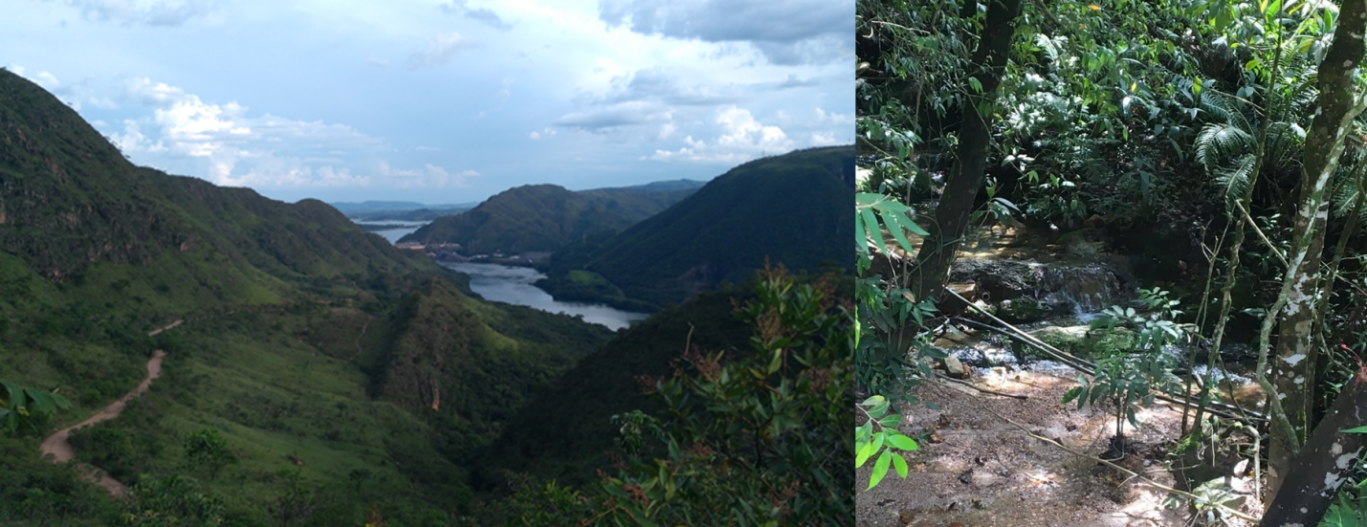


**Figure S1.** Tadpole collection site. On the left, general view showing the Peixoto dam in the background, Ibiraci municipality, Minas Gerais; on the right, semi-permanent stream during rainy month (February 2019).

**Figure S2.** The linear regression of slopes indicates the corresponding heating rates (ΔT) in the experiments for upper thermal limits (CT_max_) of acclimated group to 18°C (continuous line, circle) and 25°C (dotted line, triangle). There are no statistical differences between slopes (P=0.436). Data points represent a point in time in which temperature was taken (*i.e.*, every 5 minutes).

**Figure S3.** Total routine (A; *r*$\dot{M}O_{2}$) and maximum (B; *m*$\dot{M}O_{2}$) metabolic rates for tadpoles acclimated to 18°C (N=8) and 25°C (N=8) and exposed to different test temperatures (15, 20, 25, 30 e 34°C). Data are presented as mean ± s.e.m.


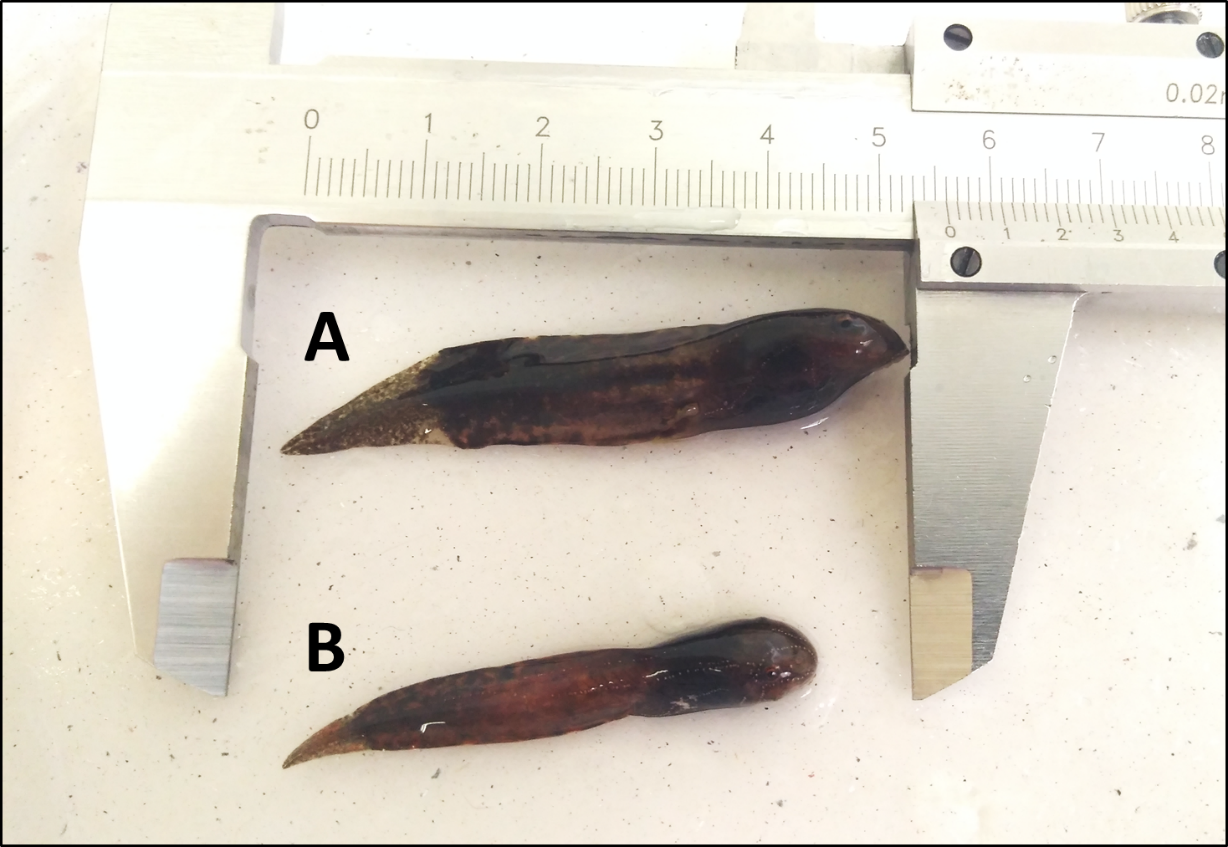


**Figure S4.** Tadpoles of *Bokermannohyala ibitiguara* from acclimated group at 18°C (A) and acclimation group at 25°C (B).

**Figure S5.** Thermal responses of heart rate (*f*_H_) and routine metabolic rate (*r*$\dot{M}O_{2}$) on tadpoles of *Bokermannohyla ibitiguara* chronically acclimated at 18° and 25°C and exposed to acute test temperatures. Continuous and dotted lines: acute thermal effect (test temperature) on *f*_H_ (A) and *r*$\dot{M}O_{2}$ (B) in tadpoles acclimated to 18 (black, circle) and 25°C (open, triangle).
